# The perception of interpersonal distance is distorted by the Müller-Lyer illusion

**DOI:** 10.1038/s41598-020-80073-y

**Published:** 2021-01-12

**Authors:** Carl Bunce, Katie L. H. Gray, Richard Cook

**Affiliations:** 1 Department of Psychological Sciences, Birkbeck, University of London, Malet Street, London, WC1E7HX UK; 2 School of Psychology and Clinical Language Sciences, University of Reading, Reading, UK

**Keywords:** Psychology, Human behaviour

## Abstract

There is growing interest in how human observers perceive social scenes containing multiple people. Interpersonal distance is a critical feature when appraising these scenes; proxemic cues are used by observers to infer whether two people are interacting, the nature of their relationship, and the valence of their current interaction. Presently, however, remarkably little is known about how interpersonal distance is encoded within the human visual system. Here we show that the perception of interpersonal distance is distorted by the Müller-Lyer illusion. Participants perceived the distance between two target points to be compressed or expanded depending on whether face pairs were positioned inside or outside the to-be-judged interval. This illusory bias was found to be unaffected by manipulations of face direction. These findings aid our understanding of how human observers perceive interpersonal distance and may inform theoretical accounts of the Müller-Lyer illusion.

## Introduction

Traditionally, social perception research has focused on the visual processing of individual faces and bodies^1–3^. In recent years, however, there has been growing interest in how human observers perceive social scenes containing multiple people^4–12^. Early findings suggest that interacting individuals may recruit regions of visual cortex that are not engaged by non-interacting individuals^12^. Similarly, social interaction displays may also recruit perceptual integration mechanisms that are not engaged by non-interacting individuals^10^. For example, where two people appear to be interacting, the facial emotion of one individual alters the perceived expression of the other^4^ and the individuals are remembered as standing closer together than they actually were^8^. These perceptual and mnemonic biases are not seen for non-interacting individuals.

Interpersonal distance is a critical cue when appraising such scenes. For instance, proxemic cues are used by observers to infer whether two people are interacting, the nature of their relationship, and the valence of their current interaction^13–15^. The inter-personal distance that interactants adopt can reveal a great deal about their attitudes towards each other. For example, people tend to distance themselves from stigmatized others, such as members of ethnic outgroups^16–18^, obese individuals^19^, and those with a disability^20^. Interpersonal distance may also provide cues to the observed individuals’ recent interaction history. For example, we tend to stand further away from people who have recently treated us unfairly^21^. Presently, however, remarkably little is known about how interpersonal distance is encoded within the human visual system.

Here we show that the perception of interpersonal distance can be distorted by the Müller-Lyer illusion; a classic optical illusion in which the distance between two points is perceived as expanded or compressed depending on the contextual information surrounding the to-be-judged interval. In conventional demonstrations, the distance between two arrow points appears to differ when the arrows point inwards and outwards^22^. Although a connecting line is a common feature in demonstrations, the illusion remains strong in its absence. Compelling demonstrations of the illusion can also be seen with other geometric forms, including diamonds. In these cases, the distance between the interior edges of two elements appears to be expanded, while the distance between the exterior edges appears compressed (for review, see Howe & Purves^23^).

To date, the Müller-Lyer illusion has primarily been studied using simple geometric forms such as arrows and diamonds^23–30^. It is currently unclear the extent to which these illusory biases present in everyday life; for example, when we view social scenes. In a series of psychophysical experiments, we demonstrate that similar illusory effects are induced when these geometric forms are replaced with pairs of human faces.

## Online testing and participant recruitment

All the experiments described were conducted online using Gorilla^31^. The use of online testing is increasingly common. Carefully-designed online tests of cognitive and perceptual processing can yield high-quality data, indistinguishable from that collected in the lab^32–34^. Participants were recruited through Prolific (https://www.prolific.co).

A sample size of 30 was chosen for each experiment. A post-hoc sensitivity analysis conducted using GPower 3.1^35^ revealed a sample of this size is sufficient to reliably detect a moderate effect size of *d*_z_ = 0.612 when conducting a paired samples *t*-test with a target power of 90% (α = 0.05, two-tailed). Participants were required to be aged 18 to 50 years-old, to have normal or corrected-to-normal visual acuity, to have no history of psychiatric or neurological illness, to reside in the United Kingdom, and to have a Prolific approval rating above 80%.

Ethical clearance was granted by the Psychological Sciences Departmental Ethics Committee at Birkbeck, University of London. The experiment was conducted in line with the ethical guidelines laid down in the 6th (2008) Declaration of Helsinki. All participants gave informed consent.

## Experiment 1

In our first and second experiment, we sought to replicate the Müller-Lyer illusion with arrows and diamonds, respectively, in order to validate our paradigm.

## Methods

**Figure 1:**
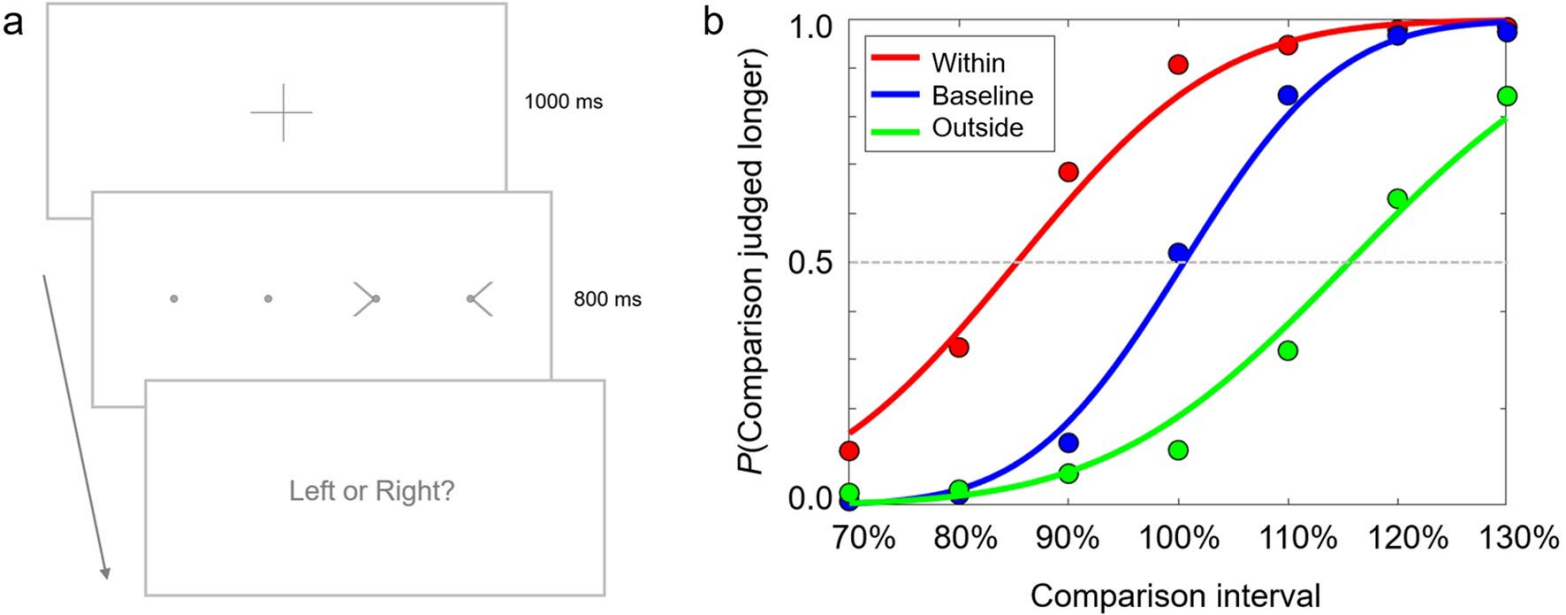
Overview of methodology. **(a)** Schematic illustration of the sequence of an experimental trial. **(b)** Participants’ binary responses were used to construct psychometric functions. The examples shown are the average functions from Experiment 4.

Thirty participants recruited via prolific (*M*_age_ = 27.23 years; *SD*_age_ = 8.30 years; 19 female) participated in Experiment 1. On each trial, participants were presented with two spatial intervals, both of which were defined as the distance between two circles. One of the intervals—the to-be-judged or ‘standard’ interval—was fixed throughout the procedure. The second interval was a comparison stimulus that varied from trial to trial. The seven comparison intervals ranged from 70% the width of the standard to 130% the width of the standard, in equidistant intervals of 10%. The two spatial intervals were presented side-by-side for 800 ms with the standard on the right and the comparison on the left (Fig. 1a). Following stimulus offset, participants had to indicate which spatial interval was greater with a keypress.

**Figure 2:**
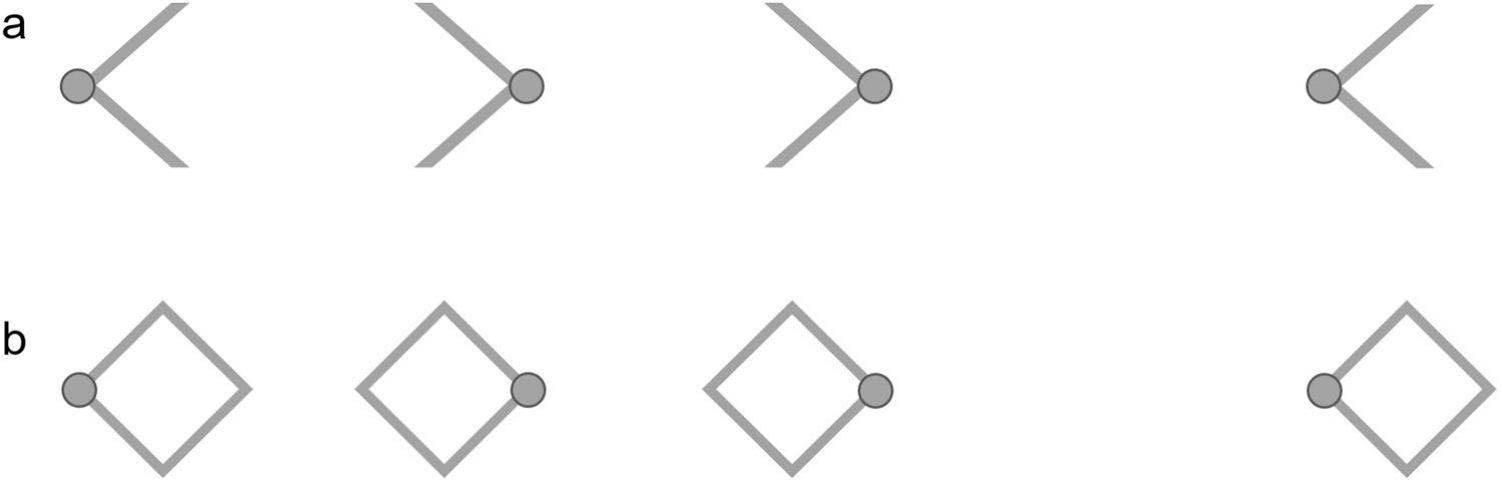
Traditional demonstrations of the Müller-Lyer illusion. The illusion is conventionally demonstrated using simple geometric forms including **(a)** arrows and **(b)** diamonds. In both cases, the target circles are the same distance apart in the left and right arrangements, but they appear closer together in the arrangements on the left.

The context element manipulation—the addition of the arrows—was applied to the standard interval only. The arrows were positioned such that their points were coincident with the target circles (Fig. 2a). In one condition the arrows were positioned outside the target circles, facing inwards. In a second condition the arrows were positioned within the target circles, facing outwards. A third condition in which no contextual information was provided served as a baseline. The width of each arrow was approximately 25% the width of the to-be-judged interval. Because the study was conducted online, we were unable to control the monitor size or the viewing distance employed by participants. Assuming a viewing distance of ~ 50 cm, we estimate that the to-be-judged interval typically subtended between 9.0° and 11.5° of visual angle. In each condition, the to-be-judged interval was presented alongside each level of comparison stimulus 20 times, yielding a total of 420 trials (7 levels of comparison × 20 presentations × 3 conditions). Trials were presented in a randomized order with breaks interspersed every 70 trials.

Participants’ binary-choice responses were used to construct psychometric functions that modelled the probability that the comparison interval was judged greater, as a function of the increasing width of the comparison interval. The perceived width of the standard interval was inferred from the point of subjective equivalence (PSE) on the resulting psychometric function (Fig. 1b). This is the hypothetical value of comparison stimulus likely to be judged identical to the standard stimulus. Psychometric functions were estimated by fitting cumulative Gaussians in Matlab (The MathWorks Inc., Natick, USA) using the Palamedes toolbox^36^. The distributions of PSE estimates in the different viewing conditions were compared using paired-samples *t*-tests (α = 0.05, two-tailed). We estimated the effect size for each comparison by calculating Cohen’s *d*_z_ (the mean difference divided by the standard deviation of the differences).

## Results

In the absence of arrows, participants judged the distance between the circles to be 97.65% (*SD* = 4.34%) of the physical distance. When inwards-pointing arrows were positioned outside the target circles, the mean distance estimate increased significantly to 116.48% (*SD* = 7.42%) [*t*(29) = 14.873, *p* < 0.001, *d*_z_ = 2.72, CI_95%_ = 1.93, 3.49]. When outwards-pointing arrows were positioned within the target circles, the mean distance estimate decreased significantly to 85.79% (*SD* = 9.82%) [*t*(29) = 9.354, *p* < 0.001, *d*_z_ = 1.71, CI_95%_ = 1.14, 2.27]. As expected, the positioning of the context elements outside the to-be-judged interval induced illusory expansion, while the positioning of the context elements within the to-be-judged interval induced illusory compression.

## Experiment 2

In our second experiment we sought to replicate the Müller-Lyer illusion with diamonds (Fig. 2b). Thirty participants were recruited via prolific (*M*_age_ = 28.47 years; *SD*_age_ = 7.16 years; 12 female). In one condition, diamonds were positioned outside the target circles, such that the circles were coincident with the interior points of the diamonds. In a second condition, they were positioned within the target circles, such that the circles were coincident with the exterior points of the diamonds. A third condition in which the contextual information was absent served as a baseline. The width of each diamond was approximately 35% the width of the to-be-judged interval.

In the absence of diamonds, participants judged the distance between the two circles to be 99.77% (*SD* = 3.48%) of the physical distance. When the diamonds were positioned outside the target circles, the mean distance estimate increased significantly to 115.66% (*SD* = 5.96%) [*t*(29) = 13.793, *p* < 0.001, *d*_z_ = 2.52, CI_95%_ = 1.78, 3.25]. When the diamonds were positioned within the target circles, the mean distance estimate decreased significantly to 83.96% (*SD* = 8.20%) [*t*(29) = 9.926, *p* < 0.001, *d*_z_ = 1.81, CI_95%_ = 1.22, 2.39].

## Experiment 3

**Figure 3:**
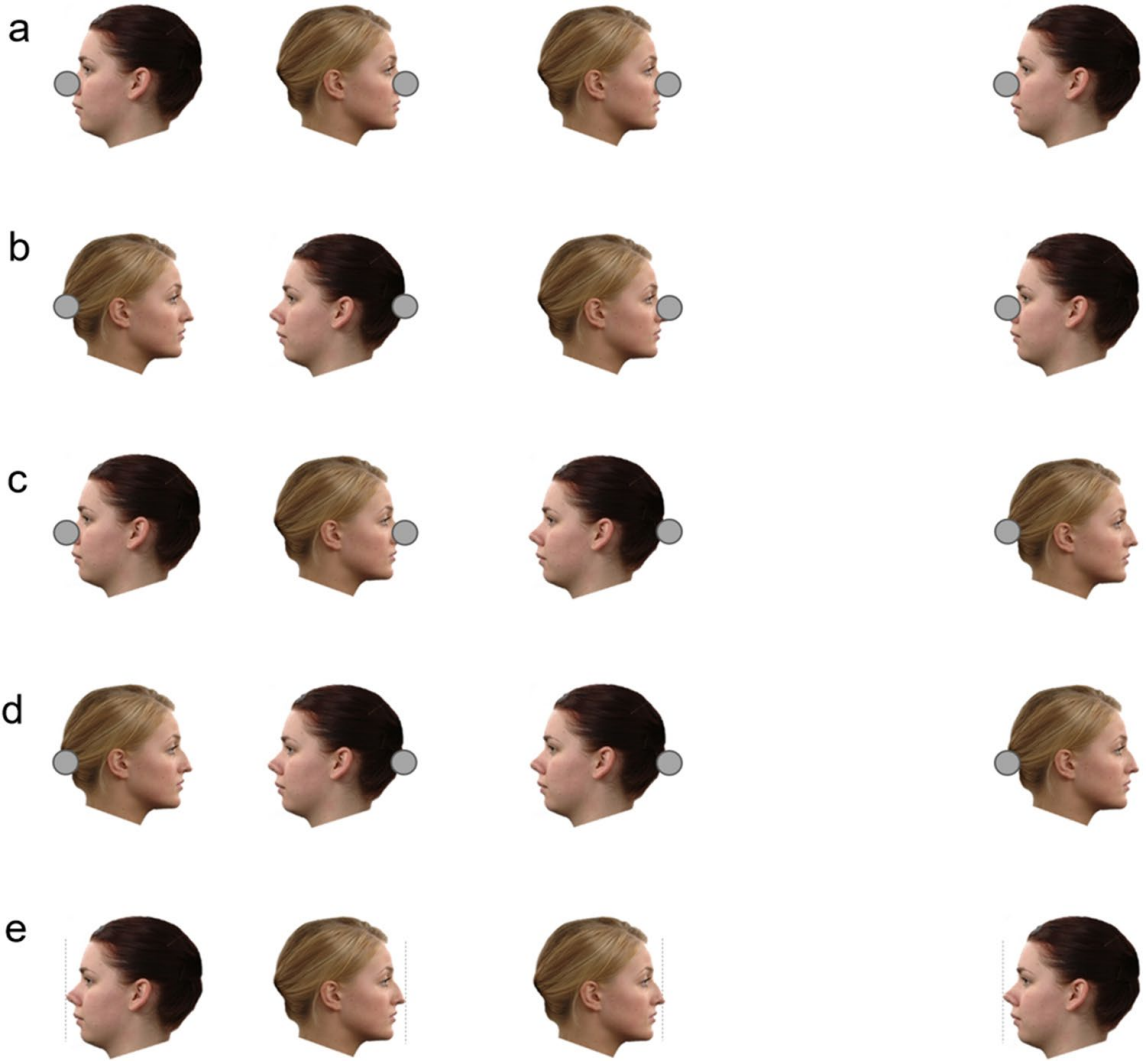
The Müller-Lyer illusion induced by faces. **(a–d)** Faces produce the illusion irrespective of their arrangement. In each case, the target circles are the same distance apart in the left and right arrangements, but they appear closer together in the arrangements on the left. **(e)** The effect manifests strongly in the absence of the target circles. The tips of the noses are equidistant in both arrangements, but appear closer in the back-to-back arrangement. Face images were taken from the Radboud Faces Database^37^.

In our third experiment we obtained identical effects when the geometric forms were replaced by faces viewed in profile (Fig. 3a). The sample (*N* = 30; *M*_age_ = 30.23 years; *SD*_age_ = 9.44 years; 16 female) included one replacement for a participant for whom we could not model psychometric functions in all conditions. Face images were taken from the Radboud Faces Database^37^, a collection of facial images that are freely available for use in academic research (http://www.socsci.ru.nl:8180/RaFD2/RaFD). The faces were positioned such that the tips of their noses were coincident with the center of the target circles. In one condition the faces were positioned outside the target circles, facing inwards. In a second condition the faces were positioned within the target circles, facing outwards. A third condition in which no contextual information was provided served as a baseline. The width of each face was approximately 35% the width of the to-be-judged interval.

In the absence of the faces, participants judged the distance between the two circles to be 100.15% (*SD* = 4.26%) of the physical distance. When two faces (arranged face-to-face) were positioned outside the target circles, the mean distance estimate increased significantly to 119.62% (*SD* = 8.00%) [*t*(29) = 14.264, *p* < 0.001, *d*_z_ = 2.60, CI_95%_ = 1.84, 3.36]. When two faces (arranged back-to-back) were positioned within the target circles, the mean distance estimate decreased significantly to 77.47% (*SD* = 11.22%) [*t*(29) = 10.894, *p* < 0.001, *d*_z_ = 1.99, CI_95%_ = 1.36, 2.61].

## Experiment 4

Next, we found that the direction of the faces made little difference; rather, the key factor was the positioning of the faces relative to the target circles. In our fourth experiment (*N* = 30; *M*_age_ = 28.97 years; *SD*_age_ = 7.28 years; 21 female), we replicated the expansion and compression effects using faces that were always arranged face-to-face (Fig. 3b). In one condition, the faces were positioned outside the target circles, such that the circles were coincident with the tips of the noses. In a second condition, they were positioned within the target circles, such that the points were coincident with the backs of the heads. A third condition in which the contextual information was absent served as a baseline.

In the absence of faces, participants judged the distance between the two circles to be 100.61% (*SD* = 5.01%) of the physical distance. When the faces were positioned outside the target circles, the mean distance estimate increased significantly to 115.47% (*SD* = 10.69%) [*t*(29) = 8.773, *p* < 0.001, *d*_z_ = 1.60, CI_95%_ = 1.05, 2.14]. When two faces were positioned within the target circles, the mean distance estimate decreased significantly to 84.13% (*SD* = 9.39%) [*t*(29) = 11.340, *p* < 0.001, *d*_z_ = 2.07, CI_95%_ = 1.43, 2.70].

## Experiment 5

In our fifth experiment (*N* = 30; *M*_age_ = 30.43 years; *SD*_age_ = 8.07 years; 16 female), we replicated the expansion and compression effects using faces that were always arranged back-to-back (Fig. 3c). In one condition, the faces were positioned outside the target circles, such that the circles were coincident with the backs of the heads. In a second condition, they were positioned within the target circles, such that the points were coincident with the tips of the noses. A third condition in which the contextual information was absent served as a baseline.

In the absence of faces, participants judged the distance between the two circles to be 100.95% (*SD* = 4.85%) of the physical distance. When the faces were positioned outside the target circles, the mean distance estimate increased significantly to 114.72% (*SD* = 8.26%) [*t*(29) = 9.147, *p* < 0.001, *d*_z_ = 1.67, CI_95%_ = 1.11, 2.22]. When two faces were positioned within the target circles, the mean distance estimate decreased significantly to 83.62% (*SD* = 7.08%) [*t*(29) = 12.893, *p* < 0.001, *d*_z_ = 2.35, CI_95%_ = 1.65, 3.05].

## Experiment 6

For completeness, we sought to confirm that the results of Experiment 3 replicate when the direction in which the faces were pointed was reversed (Fig. 3d). In one condition the faces were positioned outside the target circles, facing outwards. In a second condition the faces were positioned within the target circles, facing inwards. A third condition in which the contextual information was absent served as a baseline. The sample (*N* = 30; *M*_age_ = 29.27 years; *SD*_age_ = 9.15 years; 16 female) included two replacements for participants for whom we could not model psychometric functions in all conditions.

In the absence of faces, participants judged the distance between the two circles to be 101.56% (*SD* = 4.23%) of the physical distance. When the faces were positioned outside the target circles, the mean distance estimate increased significantly to 117.36% (*SD* = 12.49%) [*t*(29) = 6.353, *p* < 0.001, *d*_z_ = 1.16, CI_95%_ = 0.69, 1.62]. When two faces were positioned within the target circles, the mean distance estimate decreased significantly to 81.51% (*SD* = 6.78%) [*t*(29) = 14.465, *p* < 0.001, *d*_z_ = 2.64 CI_95%_ = 1.87, 3.40].

## Experiment 7

In Experiments 1–6, we asked participants to judge the distance between two target circles, and examined how these judgements were affected by the positioning of arrows, diamonds, and faces. The use of target circles was intended to help participants understand what was required of them. In our sixth experiment, however, we sought to confirm that the expansion and compression effects observed alter the perception of interpersonal distance in the absence of the target circles. The sample (*N* = 30; *M*_age_ = 30.10 years; *SD*_age_ = 9.22 years; 19 female) included three replacements for participants for whom we could not model psychometric functions in all conditions.

In the experimental conditions, participants were asked to judge the distance between the tips of the two actors’ noses (Fig. 3e). In one condition, the faces were arranged face-to-face (akin to the ‘outside’ conditions described above); in the other they were arranged back-to-back (akin to the ‘within’ conditions described above). In a baseline condition, participants judged the length of a horizontal line in the absence of any other elements. The to-be-judged distance was the same in all three conditions. The seven levels of comparison distance were defined by the length of a horizontal line.

In the baseline condition, participants judged the length of the line to be 100.84% (*SD* = 2.71%) of the physical distance. When judging the nose-to-nose distance of two faces positioned face-to-face, distance estimates (*M* = 121.32%; *SD* = 11.05%) increased significantly [*t*(29) = 10.038, *p* < 0.001, *d*_z_ = 1.83, CI_95%_ = 1.24, 2.42]. When judging the nose-to-nose distance of two faces positioned back-to-back, distance estimates (*M* = 77.64%; *SD* = 10.67%) decreased significantly [*t*(29) = 11.518, *p* < 0.001, *d*_z_ = 2.10, CI_95%_ = 1.45, 2.74].

## Experiment 8

**Figure 4:**
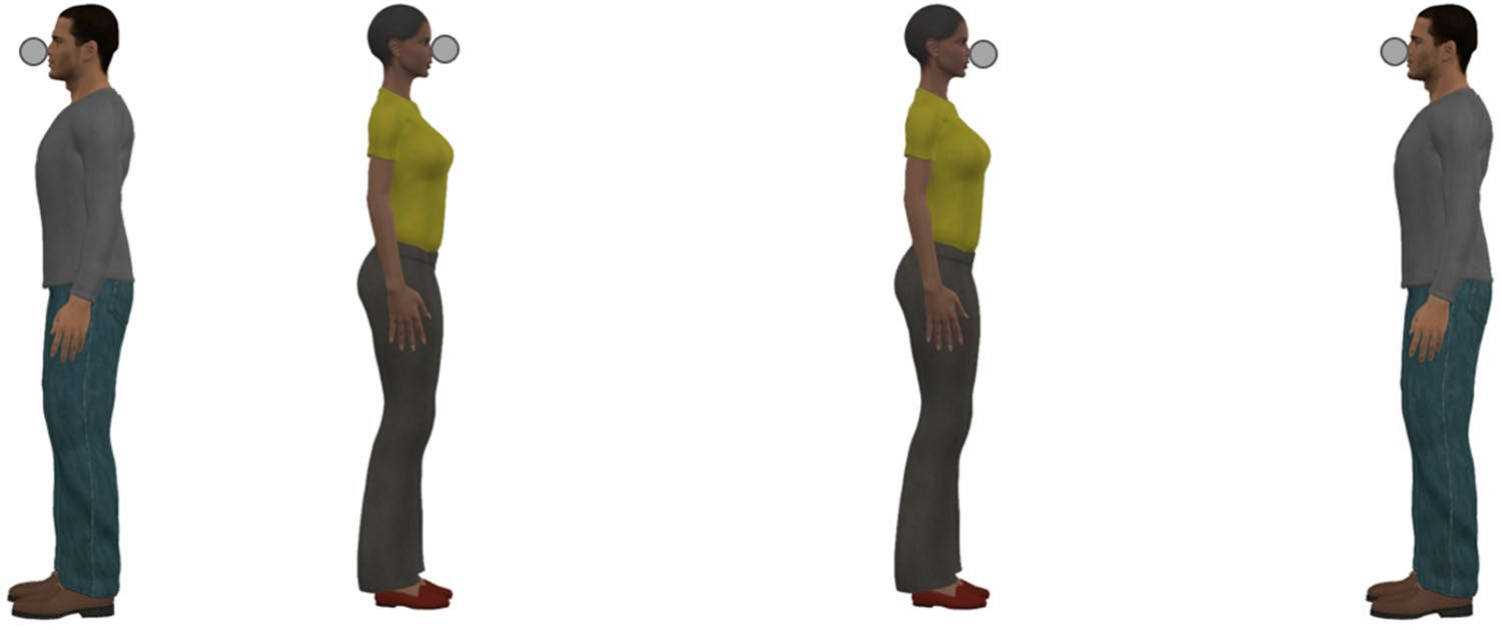
The Müller-Lyer illusion induced by whole-body stimuli. The target circles are the same distance apart in the left and right arrangement, but they appear closer together in the arrangement on the left.

In our final experiment we sought to confirm that similar effects are induced when the faces are presented in the context of whole bodies. Three-dimensional models arranged in Poser Pro 11.2 (Bondware, Inc., Murfreesboro, USA) were used as stimuli. Target dots were positioned at face level, coincident with the tips of the models’ noses (Fig. 4). The width of each model was approximately 20% of the width of the to-be-judged interval. In one condition the models were positioned outside the target circles, facing inwards. In a second condition they were positioned within the target circles, facing outwards. A third condition in which no contextual information was provided served as a baseline. The sample (*N* = 30; *M*_age_ = 29.53; *SD*_age_ = 8.84; 10 female) included two replacements for participants for whom we could not model psychometric functions in all conditions.

In the absence of the faces/bodies, participants judged the distance between the two circles to be 101.91% (*SD* = 4.94%) of the physical distance. When the faces/bodies were positioned outside the target circles, the mean distance estimate increased significantly to 105.62% (*SD* = 7.86%) [*t*(29) = 2.384, *p* = 0.024, *d*_z_ = 0.44, CI_95%_ = 0.06, 0.81]. When faces/bodies were positioned within the target circles, the mean distance estimate decreased significantly to 90.49% (*SD* = 5.18%) [*t*(29) = 10.938, *p* < 0.001, *d*_z_ = 2.00 CI_95%_ = 1.37, 2.62].

## Discussion

Interpersonal distance is a critical cue when appraising social scenes. Proxemic cues are used by observers to infer whether two people are interacting, the nature of their relationship, and the valence of their current interaction^13–15^. However, our findings reveal that judging interpersonal distance is more complex than it may first appear. Observers’ judgements may be subject to opposing biases depending on the particular distance being judged. For example, when viewing two people standing face-to-face, our findings indicate that the nose-to-nose distance between them would appear expanded; for example, a physical distance of 100 cm may appear to be closer to 105–115 cm. Conversely, when shown back-to-back, the nose-to-nose distance would appear compressed; for example, a physical distance of 100 cm may appear to be closer to 95–85 cm.

The effects of the illusory distortion described appear maladaptive and counter-intuitive. For example, when learning to dance or socially distance, a perceptual bias that distorts judgements of interpersonal distance may hinder our ability to replicate interpersonal distances modelled by others. There is currently a lack of consensus about the cause and functional significance of the Müller-Lyer illusion^23–30^. Prominent explanations have argued the illusion is a product of misapplied size constancy scaling due to misperception of embedded three-dimensional cues^28^, perceptual compromise due to conflicting local and global features^29^, or reliance on probabilistic visual processing strategies that depend on statistical regularities in the environment^23^. It is unclear whether any of these accounts can explain the version of the illusion described here.

There has been much interest in apparent cross-cultural variation in susceptibility to the Müller-Lyer illusion (for review, see Henrich et al^38^). For example, while adult observers from the US are highly sensitive to the traditional version of the Müller-Lyer illusion created with inwards and outwards facing arrows, adults from a forager community in the Kalahari showed little or no susceptibility^39^. Evidence of cross-cultural variability has led some authors to suggest that the illusion induced by simple geometric forms is a product of exposure to particular kinds of sensory input that are more common in WEIRD (Western Educated Industrialized Rich Democratic) cultures, including the corners of rooms^38,39^. Given that human faces are a ubiquitous feature of the visual environment in all human societies, it may be interesting to examine whether observers who show little susceptibility to traditional variants of the Müller-Lyer illusion, show greater susceptibility to the face variant.

It has been argued that pairs of individuals arranged face-to-face engage domain-specific perceptual processing in observers, aiding the detection and representation of social interactions^10^. Conversely, back-to-back arrangements are not thought to be processed as social interactions, and thus do not benefit from domain-specific social interaction processing. Consistent with this suggestion, front-to-front arrangements engage distinct regions of visual cortex, not recruited by back-to-back arrangements^12^. Similarly, the affective state of one individual alters the perceived emotion of another, when two people are shown face-to-face, but not back-to-back^4^.

Unsurprisingly, however, the perception of social scenes is also affected by domain-general attentional and perceptual processes. In visual search tasks, for example, pairs of individuals arranged face-to-face are found faster than pairs arranged back-to-back^6,8^. Early interpretations argued that this effect was the product of a domain-specific mechanism^6,8,10^. However, it was subsequently shown that pairs of arrows arranged point-to-point are also found faster than pairs of arrows arranged base-to-base^40^. The fact that the search advantage was replicated with arrows argues against the domain-specific view, and instead suggests that the search advantage is a product of domain-general direction cueing.

The illusory effects described here appear to be another kind of domain-general influence on the perception of social scenes. It is well established that a range of simple (non-social) geometric forms induce the Muller-Lyer illusion^23–29^. Similarly, the fact that the illusion manifests strongly irrespective of the arrangement of the faces (e.g. face-to-face, back-to-back) further argues against any explanation based on the perception of social interaction^10^. Nevertheless, authors seeking to use the back-to-back vs. face-to-face manipulation to isolate the neurocognitive mechanisms recruited by interacting (but not non-interacting) individuals, should consider how the illusory effects described here may affect their results. For example, it is possible that a spontaneous tendency to focus on the eyes and nose of the people shown, might induce an expansion effect when viewing face-to-face dyads, but a compression effect when viewing back-to-back dyads.

## Data Availability

Data for all experiments can be accessed here: https://osf.io/bswx4/.
